# Half a gram – a thousand lives

**DOI:** 10.1186/1477-7517-5-22

**Published:** 2008-06-24

**Authors:** Lev Levinson

**Affiliations:** 1 Head of the Drug Policy Program, Institute for Human Rights, Moscow, Russia

## Abstract

The article presents information on the latest drug policy change in the Russian Criminal Code: a decrease of the drug threshold amounts for which possession can lead to a criminal liability. Also, the article presents an assessment of the 2003–2004 liberal revisions in the Criminal Code, and an analysis of the background/premise for the 2006 counter-reform. The author examines the new criteria for establishing criminal liability and possible consequences of these changes for people who use illicit psycho-active substances for non-medical purposes.

## Introduction

Russian drug policy has moved a step backward: as of February 11, 2006, RF Government Decree No. 231 of May 6, 2004 is no longer in effect. For purposes of the Criminal Code, this document defined the mean single use doses of every controlled psychoactive substance. Ten to fifty times the mean dose was defined as a large amount, and a dose fifty or more times the mean was defined as an exceptionally large amount. The interpretation in note 2 to article 228 of the RF Criminal Code in revision No. 162-FL of December 8, 2003 was that the criterion used for establishing criminal liability would be the mean single dose.

Now this procedure has been revised. Federal Law No. 11-FL of January 5, 2006, which went into effect on February 11, has restored the previous model, according to which large and exceptionally large doses are defined on the basis of absolute amounts.

### Measurement of amounts of controlled psychoactive substances

Measurement of the amount of confiscated drugs is the central factor determining criminal liability for illegal drug trafficking. According to Russian laws, for acts not involving sale (acquisition, possession, transportation, production, or processing), the amount of the substance involved in the act is the sole determinant of whether the perpetrator is criminally prosecuted or is subject only to administrative punishment in the form of a fine of up to 1000 rubles or 15 days of detention. According to article 228 of the Criminal Code, violations not involving the sale of narcotics are a crime only if they involve a large amount of drugs. In other words, the higher the bar set for a large amount, the fewer criminals there will be.

The sale of narcotics, like their production for purposes of distribution, is a criminal offense regardless of amount involved. But even here amount plays a significant role. According to the revision of article 228^1 ^of the Criminal Code now in force, sale of an amount that is not large is considered a felony and is punishable by incarceration for four to eight years. Sale of narcotics in an amount defined as large or exceptionally large is a higher class of felony; for sale of a large amount the stipulated punishment is up to twelve years, while for an exceptionally large amount the punishment is up to twenty years of incarceration. With regard to acts defined as sale it is clearly very important to differentiate between the transfer of one or two doses of narcotics from one drug addict to another and the supply of a batch of narcotics weighing several kilograms.

### Background and premise for the 2003–2004 Criminal Code Revision

One of the main problems with the Criminal Code in its previous revision, which was in effect from December 8, 2003 to May 12, 2004, was the lack of proper regulation of the definition of amounts of narcotics. In practice, law enforcement agencies and courts were guided by the Summary Table from the Expert Report of the International Narcotics Control Board, which has become infamous as the "Babayan Table" (named for the permanent chairman of this institution). This table is a recommendation published by a scientific consulting agency without either official registration or legally approved status, which is not and could not be authorized to participate in the adoption of regulations. In spite of this, the table has been used in all criminal cases without exception (i.e., in hundreds of thousands of cases annually). According to this table, a large amount is considered to be, for example, 0.1 g of marijuana, and 0.005 g of heroin is considered an exceptionally large amount. This approach to amounts made it possible, on the basis of article 228 of the Criminal Code (the version in effect at that time) to hold those caught with 0.0005 g and 100 kg of heroin equally liable to a penalty of 7 to 15 years of incarceration. When this table was in general use, those sentenced to long periods of incarceration were usually young people who had acquired, possessed, sold or distributed hundredths of a gram, while the "sharks of the narcotics business" remained outside the purview of law enforcement agencies. This single table was used to determine the criminal liability of both classes of offenders.

The 2003 revisions to the Criminal Code in 2003 were intended to put an end to this invidious practice. In general, they succeeded. Although the mean single doses hurriedly established in spring 2004 had not been considered in sufficient depth for all the substances on the list (as is clear in the appended comparative table), for the most widely used substances – heroin, marijuana, amphetamines, cocaine – the doses established by the May 2004 Decree were confirmed to be realistic street doses (instead of the arbitrary pharmacological doses in the Babayan Table), so that it was less likely that quantities that were actually small would be defined as large amounts.

As a result, after the new, significantly increased threshold amounts went into effect, approximately 40,000 people who had previously been convicted were freed or had their sentences decreased significantly, and in 2004–2005 a minimum of 60,000 individuals who, had it not been for the 2003 revision, would have been tried as criminals under the article concerning possession or acquisition without intent to sell, avoided criminal prosecution.

### Causes for 2003 regressive revisions

Naturally, such a successfully implemented reform delivered a blow to vested interests, especially those of the Federal Service to Control Narcotics Trafficking. The Narcotics Police Force, despite glowing reports, is in a very bad position; the 40,000-person force cannot justify the resources squandered on it. With one and a half criminal cases per agent brought annually (and the overwhelming majority of these cases are simple ones at that) more than 12 billion rubles a year is an unacceptable luxury. This was discussed at an expanded session of the staff of the Prosecutor General's Office on February 3, 2006, by Prosecutor General Vladimir Ustinov.

"With regard to numbers of personnel, this is the largest such service in the world. However, if only the quality were comparable to the quantity. Drug control agents were responsible for bringing to light only one third of the total number of crimes (prosecuted). The remainder continues to have been the work of the police and other law enforcement agencies. Among completed investigations of criminal cases only one in four were performed by these drug control agents. And their cases took the longest and had the lowest quality investigations. As for the nature of the crimes they brought to light, then as before, they are predominantly those that are superficial. These are crimes of acquisition, possession, transport, and processing of narcotics. But they rarely bring to light crimes that are closer to the root of the matter, those with which the narcotics chain begins and that enable the acquisition, possession, production, and transport of narcotics. In the entire year, they brought to light only 124 instances of illegal production of narcotics, 371 cases of inducement to drug addiction, and only a little more than 3,000 cases involving setting up or maintaining narcotics dens."

Under these circumstances, threshold amounts became a survival issue for the Federal Service to Control Narcotics Trafficking.

Despite the fact that the concept of a mean single dose was proposed in 2003 by the President of the Russian Federation and that, in public speeches of that time, the President argued for the need to differentiate between approaches to drug users and to drug traffickers, the Federal Service to Control Narcotics Trafficking managed to turn the tide in its favor. On May 6, 2005, giving in to pressure from the Drug Control Service, the Government submitted a draft law to the State Duma that excluded the criteria of mean single doses from article 228 of the Criminal Code.

The result of this retreat was a change in the Criminal Code and adoption of RF Government Decree No. 76 of February 7, 2005, "On confirming large and exceptionally large amounts of narcotics and psychotropic substances for the purposes of articles 228, 228^1 ^[1] and 229 of the Russian Federation Criminal Code."

### Analysis of 2006 policy revisions

Now, when the regressive revision of criminal drug policy has been completed and when, based on the new rules, new amounts have been approved for defining large and exceptionally large amounts, two conclusions can be drawn on the basis of the figures:

1) Since February 11, 2006 there are additional grounds for the criminal prosecution of people who use narcotics.

2) Despite the most recent changes, compared with the situation existing until May 12, 2004, today's position does not seem to be as universally repressive as it was during the period when Academician Babayan's Summary Table was in use.

This can be confirmed by a comparative analysis of the threshold of criminal liability with respect to the most important substances on the list (table 1).

**Table 1:** Amounts of narcotics and psychotropic substances deemed to be large and exceptionally large amounts (in grams)

Substances	Babayan Table as of March 1, 2003		Decree of May 6, 2004		Decree of February, 7, 2006	
	**large amount**	**exceptionally large amount**	**large amount**	**exceptionally large amount**	**large amount**	**exceptionally large amount**

Cannabis (Marijuana)	0.1	500	20	100	6	100
Hashish	0.1	100	5	25	2	25
Cannabis oil (Hashish oil)	0.05	50	1	5	0.4	5
Heroin	any amount up to 0.005	0.005	1	5	0.5	2.5
Opium	0.1	10	5	25	1	25
Acetylated opium	0.05	5	1	5	0.5	5
Poppy straw	0.2	250	100	500	20	500
Poppy straw extract (poppy straw concentrate)	0.02	2	0.5	2.5	1	5
Methadone	0.01	1	0.5	2.5	0.5	2.5
Buprenorphine	0.0012	0.12	0.003	0.015	0.005	0.025
Morphine	0.01	1	0.1	0.5	0.1	0.5
Codeine	0.2	10	1	5	1	5
Ketamine	0.02	1	1	5	0.2	5
3-Methylfentanyl	any amount up to 0.002	0.002	0.0002	0.001	0.0002	0.001
Sodium oxybutyrate and other salts of Hydroxybutyric acid	25	250	20	100	10	50
Pentazocine	0.03	3	0.5	2.5	2	10
Trimeperidine (Promedol)	0.03	3	0.2	1	0.03	0.15
Cocaine	0.01	1	1.5	7.5	0.5	5
Cocaine hydrochloride	0.01	1	0.1	0.5	0.5	5
Home made derivatives of Ephedrine or substances containing Ephedrine	1 ml	100 ml	3	10	0.5	10
LSD	any amount up to 0.0001	0.0001	0.003	0.015	0.0001	0.005
Mescaline	0.03	5	0.5	2.5	0.5	2.5
MDMA ("Ecstasy")	0.02	1	0.5	2.5	0.6	3
Methamphetamine, Pervitine	0.02	1.5	0.5	2.5	0.3	2.5
Amphetamine (Phenamine) and combined medicinal drugs containing Phenamine Amphetamine)	0.03	3	1	5	0.2	1
Fruit body (any part) of any species of mushroom containing Psilocibin and/or) Psilocin	0.5	50	only the pure substance was counted	only the pure substance was counted	10	100
Psilocibin, Psilocin	0.01	0.1	0.05	0.25	0.05	0.25
Phencyclidine	any amount up to 0.01	0.01	0.05	0.25	0.02	0.1
Ephedrine (Methcathinone)	0.02	3	0.5	2.5	0.2	2.5
Cathinone	0.02	1	0.005	0.025	0.2	1
Amobarbital (Barbamyl)	0.6	30	1	5	1	5
Glutethimide (Noxyron)	1.5	25	2.5	12.5	1	12.5
Methaqualone	0.05	1	2	10	1	5
Taren	10	100	2	10	0.5	10

If we consider the whole list, containing 232 items, we find that a decrease in amounts defined as large occurred in only 49 cases, while there were increases in 140. These amounts were increased in the majority of listed substances.

However, almost all of the 140 substances for which the amount deemed large was increased are substances only rarely involved in illegal trafficking, while decreases in threshold occurred for the majority of popular narcotics.

The bar for the amount criminally punishable decreased with respect to that established on May 12, 2004: by a factor of two for heroin (from 1 to 0.5), by a factor of five for opium (from 5 to 1 grams), for marijuana from 20 to 6 grams, for hashish from 5 to 2 grams, for ketamine by a factor of 5 (from 1 to 0.2 grams), for cocaine by a factor of three (from 1.5 to 0.5 grams), for LSD by a factor of 30 (from 0.003 to 0.0001 gram), for pervitine from 0.5 to 0.3 grams, for amphetamine by a factor of 5 (from 1 to 0.2 grams), and for ephedrine from 0.5 to 0.2 grams.

The situation was analogous with regard to amounts defined as exceptionally large. These amounts were increased for 140 substances, while they decreased for 29. But here too, although in a smaller number of cases, the criminal penalty was increased for the most widely distributed drugs, especially for heroin, an exceptionally large amount of which was decreased by a factor of two (from 5 to 2.5 grams), and also for cocaine, promedol, and LSD.

For all substances included in List 1 (in particular, cannabis and its derivatives, heroin, opium, poppy straw, methadone, MDMA, phencyclidine, ephedrine, cathinone, and methqualone), the large and exceptionally large amounts are again weighed and defined with impurities included, regardless of their percentage admixture. This also applied to Babayan's summary table. The same rule was established for cocaine (in List II). In the May 12, 2004 Decree there was no such rule, justifying insistence on measurement of the weight of the pure amount of controlled substance in the mixture. It is true that for heroin, in the majority of cases, despite the letter of the law, this was not possible. (Investigators and experts attributed this to the lack of the necessary equipment for extracting the diacetylmorphine from street heroin.)

## Conclusion

Decrease in the threshold entails completely different penal consequences for different substances. The change in amount requiring criminal penalties for marijuana from 20 to 6 grams will not lead to a significant overall increase in the number of those subject to criminal prosecution, since 5.99 grams of marijuana is still a sufficient quantity for more than one use. However, the difference in threshold between a gram and half a gram of heroin will cause many drug dependent people to be incarcerated. What is significant here is not just that quality of heroin (additives mixed with the diacetylmorphine) affects its weight, but, most important, that heroin addiction requires a higher dose for more frequent use. Given the current level of heroin use, which, although decreasing, is still very high, the number of people affected by this single line in the table is very high. And this will increase the number of people in prison and contribute to a new round of growth in the prison population.

A Russian translation of the text may be read in Additional file 1.

## Competing interests

The author declares that they have no competing interests.

## Supplementary Material

Additional file 1 ПОлгРамма – и тысячи судеб. Russian translation of Half a gram – a thousand lives Click here for file
