# Supplementary material for: Half a gram – a thousand lives
Source: Harm Reduct J. 2008 Jun 24;5:22. doi: 10.1186/1477-7517-5-22 (PMC2474596; doi:10.1186/1477-7517-5-22)
Supplement: Additional file 1 — ПОлгРамма – и тысячи судеб. Russian translation of Half a gram – a thousand lives [file 1477-7517-5-22-S1.doc]

# Полграмма – и тысячи судеб

Лев Левинсон,

программа «Новая наркополитика»

Российская наркополитика дала обратный ход: 11 февраля 2006 года утратило силу постановление Правительства РФ от 6 мая 2004 года № 231. Этим документом для целей Уголовного кодекса определялись средние разовые дозы потребления по каждому из запрещенных к свободному обороту психоактивных веществ. От десяти доз признавалось крупным размером, от пятидесяти – особо крупным. Такова была конструкция примечания 2 к статье 228 УК РФ в редакции Федерального закона от 8 декабря 2003 года № 162-ФЗ: критерием, применяемым для установления уголовной ответственности, служили средние разовые дозы.

Теперь этот порядок изменен. Федеральным законом от 5 января 2006 года № 11-ФЗ, вступившим в силу 11 февраля, восстановлена существовавшая ранее модель, по которой крупный и особо крупный размеры определялись в абсолютных величинах.

Определение размера изъятых наркотиков – основной элемент всей системы уголовной ответственности за их незаконный оборот. В отношении деяний, не связанных со сбытом (приобретение, хранение, перевозка, изготовление, переработка), только от размера проходящего по делу вещества зависит, подвергнется ли нарушитель уголовному преследованию за совершение перечисленных поступков либо наказание будет административным в виде штрафа до 1000 рублей или 15 суток ареста. Согласно статье 228 УК правонарушения, не связанные со сбытом наркотиков, оказываются преступлениями только в случае, если они совершены в крупном размере. То есть, чем выше планка крупного размера, тем меньше преступников.

Сбыт наркотиков, равно как их производство в целях распространения, уголовно наказуемы в любом случае, независимо от количества. Но и здесь размер играет существенную роль. По действующей редакции статьи 228^1^ УК продажа в количестве, не являющемся крупным, считается тяжким преступлением и наказывается лишением свободы на срок от 4 до 8 лет. Сбыт же в размере, признаваемом крупным либо особо крупным – особо тяжкие преступления (за крупный размер предусмотрено до 12, а за особо крупный – до 20 лет лишения свободы). Очевидно, что и в отношении действий, квалифицируемых как сбыт, дифференциация очень важна: одно дело – передача одним наркоманом другому одной-двух доз, другое – поставка партии наркотиков весом в несколько килограмм.

Одной из основных проблем УК в его прежней, действовавшей до 12 мая 2004 года (дата вступления в силу закона от 8 декабря 2003 года) редакции, была неурегулированность вопроса об определении размера наркотиков. На практике правоохранительные органы и суды руководствовались Сводной таблицей заключений Постоянного комитета по контролю наркотиков, печально известной в народе как «таблица Бабаяна» (по имени бессменного председателя этого учреждения). Таблица представляла собой рекомендательный документ, изданный не имеющим государственной регистрации и законодательно утвержденного статуса научно-консультативным органом, в компетенцию которого не входило и не могло входить принятие нормативных актов. Несмотря на это, таблица применялась по всем без исключения уголовным делам (т.е. по сотням тысяч дел ежегодно). Согласно таблице, крупным размером признавалось, например, 0,1 г марихуаны, особо крупным размером – 0,005 г героина. Такой подход к количествам позволял и за 0,005 г и за 100 кг героина привлекать по части четвертой статьи 228 УК (в действовавшей тогда редакции) к равной ответственности – от 7 до 15 лет лишения свободы. В массовом порядке к длительным срокам лишения свободы приговаривались молодые, как правило, люди, приобретшие, хранившие или продавшие/передавшие сотые доли грамма, тогда как «акулы наркобизнеса» оставались вне поля зрения правоохранительных органов. Отчетность и по тем, и по другим велась по одной графе.

Столь порочную практику, порождавшуюся несовершенством законодательства, призваны был остановить изменения, внесенные в УК в 2003 году. И, в основном, это удалось. Хотя подготовленные в спешке весной 2004 года размеры средних разовых доз не были достаточно глубоко проработаны для всех веществ списков (что видно из прилагаемой сопоставительной таблицы), по наиболее распространенным позициям – героину, марихуане, амфетаминам, кокаину – постановлением от 6 мая 2004 года были утверждены реалистичные уличные дозы (вместо бабаяновских условных фармакологических), позволившие уйти от оценки любого количества как якобы крупного размера.

В результате после вступления в силу новых значительно увеличенных размеров около 40 тысяч ранее осужденных было освобождено или получило существенное сокращение срока наказания, а за 2004 – 2005 годы благодаря «дозам» смогли избежать уголовного преследования как минимум 60 тысяч человек, которые, не будь поправок 2003 года, получили бы статью за хранение или приобретение без цели сбыта.

Естественно, что столь успешно проведенная реформа ударила по ведомственным интересам, прежде всего – по интересам Федеральной службы по контролю за оборотом наркотиков (ФСКН). Наркополиция, несмотря на бодрые отчеты, находится в бедственном положении. 40-тысячное воинство не оправдывает разбазариваемых на него ресурсов. При полутора уголовных делах в год на сотрудника (да и делах-то, в подавляющем большинстве, несложных) более 12 миллиардов рублей в год – непозволительная роскошь.

Об этом говорил на расширенном заседании коллегии Генеральной прокуратуры 3 февраля 2006 года Генпрокурор Владимир Устинов:

«По численности эта служба самая большая в мире. Но, если бы количество сопровождалось соответствующим качеством. Сотрудниками органов наркоконтроля выявляется только треть общего количества преступлений. Остальные по-прежнему приходятся на милицию и другие правоохранительные органы. Среди оконченных расследованием уголовных дел лишь каждое четвёртое – дело наркоконтроля. По делам самые длительные сроки и самые низкие показатели качества следствия. Что же касается характера выявляемых преступлений, то среди них, как и прежде, больше тех, которые находятся на поверхности. Это приобретение, хранение, изготовление, перевозка наркотиков. А вот преступления глубинного характера, с которых начинается наркоцепочка и которые продуцируют приобретение, хранение наркотиков, выявляются редко. За весь год выявлено всего 124 случая незаконного производства наркотиков, 371 - склонения к потреблению наркотиков и чуть больше 3 тыс. случаев организации либо содержания притонов.»

При таких обстоятельствах вопрос о размерах стал для ФСКН вопросом выживания.

Несмотря на то, что концепция средних разовых доз была предложена в 2003 году Президентом РФ, и в публичных вступлениях того времени Президент отстаивал необходимость дифференцированного подхода к наркоманам и наркоторговцам, ФСКН переломила ситуацию в свою пользу. 6 мая 2005 года, поддавшись давлению наркоконтроля, Правительство внесло в Государственную Думу законопроект об исключении из статьи 228 УК критерия средней разовой дозы.

Итог этого отступления – изменение УК и принятие постановления Правительства РФ от 7 февраля 2006 года № 76 «Об утверждении крупного и особо крупного размеров наркотических средств и психотропных веществ для целей статей 228, 228^1^ и 229 Уголовного кодекса Российской Федерации».

Теперь, когда процесс возвратного пересмотра уголовной наркополитики завершен, когда по новым правилам утверждены новые количества для определения крупного и особо крупного размеров, можно, основываясь на цифрах, сделать два вывода:

1) с 11 февраля 2006 года для уголовного преследования людей, употребляющих наркотики, появились дополнительные основания;

2) по сравнению с ситуацией, существовавшей до 12 мая 2004 года, нынешнее положение все равно, несмотря на последние изменения, представляется не столь всеобъемлюще репрессивным, каковым оно было в эпоху применения Сводной таблицы академика Бабаяна.

Это подтверждает сравнительный анализ порога уголовной ответственности по основным веществам списка.

Таблица

Количества наркотических средств и психотропных веществ, признаваемые крупным и особо крупным размерами (в граммах), в извлечениях

| вещество | Таблица Бабаяна  по состоянию на 1 марта 2003 года | | Постановление от 6 мая 2004 года | | Постановление от 7 февраля 2006 года | |
| --- | --- | --- | --- | --- | --- | --- |
|  | **крупный размер** | **особо крупный**  **размер** | **крупный размер** | **особо крупный** | **крупный** | **особо крупный** |
| каннабис (марихуана) | 0,1 | 500 | 20 | 100 | 6 | 100 |
| гашиш | 0,1 | 100 | 5 | 25 | 2 | 25 |
| масло каннабиса (гашишное масло) | 0,05 | 50 | 1 | 5 | 0,4 | 5 |
| героин | любое количество до 0,005 | 0,005 | 1 | 5 | 0,5 | 2,5 |
| опий | 0,1 | 10 | 5 | 25 | 1 | 25 |
| ацетилированный опий | 0,05 | 5 | 1 | 5 | 0,5 | 5 |
| маковая солома | 0,2 | 250 | 100 | 500 | 20 | 500 |
| экстракт маковой соломы (концентрат маковой соломы) | 0,02 | 2 | 0,5 | 2,5 | 1 | 5 |
| метадон | 0,01 | 1 | 0,5 | 2,5 | 0,5 | 2,5 |
| бупренорфин | 0,0012 | 0,12 | 0,003 | 0,015 | 0,005 | 0,025 |
| морфин | 0,01 | 1 | 0,1 | 0,5 | 0,1 | 0,5 |
| кодеин | 0,2 | 10 | 1 | 5 | 1 | 5 |
| кетамин | 0,02 | 1 | 1 | 5 | 0,2 | 5 |
| 3-метилфентанил | любое количество до 0,002 | 0,002 | 0,0002 | 0,001 | 0,0002 | 0,001 |
| натрий оксибутират и другие соли оксимасляной кислоты | 25 | 250 | 20 | 100 | 10 | 50 |
| пентазоцин | 0,03 | 3 | 0,5 | 2,5 | 2 | 10 |
| тримеперидин (промедол) | 0,03 | 3 | 0,2 | 1 | 0,03 | 0,15 |
| кокаин | 0,01 | 1 | 1,5 | 7,5 | 0,5 | 5 |
| кокаина гидрохлорид | 0,01 | 1 | 0,1 | 0,5 | 0,5 | 5 |
| кустарно приготовленные препараты из эфедрина или из препаратов, содержащих эфедрин | 1 мл | 100 мл | 3 | 10 | 0,5 | 10 |
| ЛСД | любое количество до 0,0001 | 0,0001 | 0,003 | 0,015 | 0,0001 | 0,005 |
| мескалин | 0,03 | 5 | 0,5 | 2,5 | 0,5 | 2,5 |
| МДМА («экстази») | 0,02 | 1 | 0,5 | 2,5 | 0,6 | 3 |
| метамфетамин, первитин | 0,02 | 1,5 | 0,5 | 2,5 | 0,3 | 2,5 |
| амфетамин (фенамин) и комбинированные лекарственные препараты, содержащие фенамин (амфетамин) | 0,03 | 3 | 1 | 5 | 0,2 | 1 |
| плодовое тело (любая часть) любого вида грибов, содержащих псилоцибин и (или) псилоцин | 0,5 | 50 | учитывалось только чистое вещество | учитывалось только чистое вещество | 10 | 100 |
| псилоцибин, псилоцин | 0,01 | 0,1 | 0,05 | 0,25 | 0,05 | 0,25 |
| фенциклидин | любое количество до 0,01 | 0,01 | 0,05 | 0,25 | 0,02 | 0,1 |
| эфедрон (меткатинон) | 0,02 | 3 | 0,5 | 2,5 | 0,2 | 2,5 |
| катинон | 0,02 | 1 | 0,005 | 0,025 | 0,2 | 1 |
| амобарбитал (барбамил) | 0,6 | 30 | 1 | 5 | 1 | 5 |
| глютетимид (ноксирон) | 1,5 | 25 | 2,5 | 12,5 | 1 | 12,5 |
| метаквалон | 0,05 | 1 | 2 | 10 | 1 | 5 |
| тарен | 10 | 100 | 2 | 10 | 0,5 | 10 |

Если посмотреть и просчитать весь перечень, включающий 232 именования, можно убедиться, что снижение крупных размеров произошло только по 49 позициям, а по 140 – по формальному большинству веществ, они были увеличены.

Однако почти все из 140, размер которых был увеличен, – редкие в незаконном обороте вещества, тогда как снижение порога произошло по большинству наиболее популярных наркотиков.

Планка наказуемости снизилась по сравнению с установленной 12 мая 2004 года, по героину в два раза (с 1 до 0,5 грамма), по опию в пять раз (с 5 до 1 грамма), по марихуане – с 20 до 6 грамм, по гашишу – с 5 до 2 грамм, по кетамину – в пять раз (с 1 до 0,2 грамма), по кокаину – в три раза (с 1,5 до 0,5 грамма), по ЛСД – в тридцать раз (с 0,003 до 0,0001 грамма), по первитину – с 0,5 до 0,3 грамма, по амфетамину – в пять раз (с 1 до 0,2 грамма), по первитину – с 0,5 до 0,3 грамма, по эфедрону – с 0,5 до 0,2 грамма.

То же самое по особо крупным размерам. Их увеличение произошло также по 140 позициям, снижение - по 29 пунктам. Но и здесь, хотя и в меньшем числе случаев, уголовная репрессия расширена по распространенным веществам, прежде всего – по героину, особо крупный размер которого сокращен в два раза (с 5 до 2,5 грамм), а также по кокаину, промедолу, ЛСД.

По всем веществам, входящим в Список I (в частности, это каннабис и его производные, героин, опий, маковая солома, метадон, ЛСД, МДМА, фенциклидин, эфедрон, катинон, метаквалон), крупный и особо крупный размеры вновь определяются с учетом примесей, независимо от их процентного содержания в смеси. Соответствующее примечание имелось и к Сводной таблице Бабаяна. То же правило восстановлено и в отношении кокаина (находящегося в Списке II). В постановлении от 12 мая 2004 года такого примечания не было, что давало основания настаивать на определении веса чистого запрещенного вещества в смеси. Правда, по героину в большинстве случаев это, вопреки букве нормативного акта, не удавалось: следователи и эксперты ссылались на отсутствие необходимого оборудования для экстрагирования диацетилморфина из уличного героина.

Понижение порога по тем или иным веществам повлечет совершенно различные карательные последствия. Если изменение размера наказуемости по марихуане с 20 до 6 грамм в целом не приведет к существенному росту привлекаемых к уголовной ответственности, поскольку 5,99 грамма марихуаны – все-таки достаточное количество для неоднократного употребления, то между граммом и полграмма героина помещается множество наркозависимых. Значение здесь имеет не только то, что «разбодяженность» героина влияет на его вес, но, прежде всего, то, что системному героиноману требуется большая доза для более частого потребления. Учитывая хотя и сокращающуюся, но все же весьма высокую распространенность героина, человекоемкость одной лишь этой позиции весьма велика. И это человекоемкость тюрьмы, вклад в новый виток роста тюремного населения.
